# Gonadal Failure in a Male With 3-M Syndrome

**DOI:** 10.1210/jcemcr/luae084

**Published:** 2024-06-06

**Authors:** Irena Aldhoon-Hainerova, Elizabeth Baranowski, Esther Kinning, Renuka P Dias

**Affiliations:** Department of Children and Adolescents, Faculty Hospital Kralovske Vinohrady, Third Faculty of Medicine, Charles University, 100 34 Prague 10, Czech Republic; Department of Paediatric Endocrinology and Diabetes, Birmingham Women´s and Children´s Hospital, B4 6NH Birmingham, UK; Institute of Metabolism and Systems Research, University of Birmingham, B15 2TT Birmingham, UK; Department of Clinical Genetics, Birmingham Women´s and Children´s Hospital, B15 2TG Birmingham, UK; Department of Paediatric Endocrinology and Diabetes, Birmingham Women´s and Children´s Hospital, B4 6NH Birmingham, UK; Institute of Metabolism and Systems Research, University of Birmingham, B15 2TT Birmingham, UK

**Keywords:** 3-M syndrome, short stature, CUL7 gene pathogenic variant, recombinant human growth hormone, hypergonadotropic hypogonadism

## Abstract

OMIM 273750 (3-M) syndrome is a rare cause of severe short stature with variable dysmorphic features caused by pathogenic variants in several genes including cullin7 gene (*CUL7*). Hypogonadism and hypospadias have been described in only a few males. We report a patient with *CUL*7 pathogenic variant who had bifid scrotum and perineal hypospadias at birth. He entered puberty spontaneously at age 12 years and appropriately completed pubertal development by 15 years. Subsequently, a regression of testicular volumes, increased gonadotropin levels, and reduced (although normal) testosterone levels were observed. This case highlights the importance of careful pubertal monitoring as pubertal dysfunction may be associated with 3-M syndrome.

## Introduction

OMIM 273750 (3-M syndrome) is a rare autosomal recessive condition caused by homozygous or compound heterozygous pathogenic variants in genes encoding cullin 7 (*CUL7*; type 1: MIM *609577), obscurin-like 1 (*OBSL1*; type 2: MIM *612921), or coiled-coil domain containing 8 (*CCDC8*; type 3: MIM *614205) (1). Pathogenic variants in the *CUL7* gene are the most frequently reported cause of this syndrome. Obscurin-1 and Cullin-7 are proteins involved in the cell machinery of the ubiquitin-proteasome pathway, which degrades unwanted proteins. Pathogenic variants in the encoding genes result in interference in the process of tagging proteins for degradation and subsequent impact on cell division and growth and response to GH.

The physical findings of 3-M syndrome are variable, and no genotype-phenotype correlations have been proven (2, 3). The described phenotype for affected individuals includes severe pre- and postnatal growth restriction (usually <−4 SD) with variable dysmorphic features including craniofacial abnormalities, such as a large head for their body height, a prominent forehead with a triangular face, and a pointed chin. Other features can include a short broad neck, prominent heels, joint hypermobility, and hyperlordosis (2). Characteristic radiological features include slender long bones and tall vertebral bodies (2).

Children with pathogenic variants in *CUL7* are significantly shorter than those with either *OBSL1* or *CCDC8* pathogenic variants (1). In affected patients, levels of IGF-1 are normal or low, and levels of serum GH in stimulation tests are usually normal. Complete or partial GH deficiency has been shown in some cases (1, 4). This may partly be explained by GH and/or IGF-1 resistance (1) and by a low expression and secretion of IGF-2 from 3-M fibroblasts (5). All these features suggest a degree of resistance in the GH-IGF axis. Growth response to recombinant human GH (rhGH) therapy is variable but typically poor (1, 4, 6).

No other significant system disorders have so far been described (2). In contrast to females, hypogonadism and hypospadias have been described in a few male patients (2, 7). Because of the small number of reported cases with 3-M syndrome, it is not clear what the incidence of gonadal dysfunction is in this context. We present a case of a male with a *CUL7* pathogenic variant who underwent an unsuccessful trial of rhGH treatment and showed pubertal arrest with evidence of hypergonadotrophic hypogonadism.

## Case Presentation

**Figure 1.**
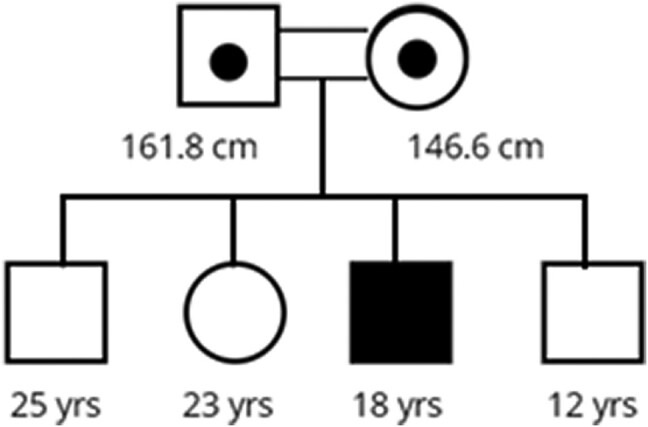
Family tree. Parental heights and current ages of all 4 siblings, including the index case (shaded black).

The patient is now an 18-year-old male who was first reviewed for short stature when he was 18 months old. Parents are a first cousin consanguineous pairing from the Mirpur region of Pakistan and are both short (paternal height 161.8 cm, maternal height 146.6 cm) but have no overt dysmorphism or disproportion. Mid-parental height is 161.8 cm (−2.02 SD score [SDS]). All siblings are of normal stature and reported to be well with no medical concerns (see Fig. 1 for the family tree).

Antenatal scans at 33 weeks suggested short limbs and trunk. He was born at 36 + 6 weeks´ gestation by emergency cesarean section because of fetal tachycardia. His birth weight was 2 kg (2nd percentile for age, sex, and gestation; −2.1 SDS), birth length was unavailable and head circumference was 34 cm (72nd percentile; 0.6 SDS). At birth, the patient was noted to have short limbs, a short trunk, and a bifid scrotum with perineal hypospadias.

He presented for the first time to our endocrine clinic at age 18 months with a height of 60.2 cm (−8.2 SDS), a weight of 6.15 kg (−5.1 SDS), and body mass index (BMI) of 17 kg/m^2^ (74th percentile; 0.6 SDS). At the time of referral, he had shown persistent severe failure to thrive. There were no concerns about his hearing, vision, or social development. The patient had profound short stature, macrocephaly (head circumference 47 cm; −0.3 SDS) with a prominent occiput and a degree of frontal bossing, depressed nasal bridge, and mid-face crowding. His stature was proportionate but with a significant lumbar lordosis. He had perineal hypospadias with a hooded prepuce, significant chordee, and a bifid scrotum, but no associated micropenis. Testes were descended and palpable bilaterally in the hemi-scrota.

## Diagnostic Assessment

**Figure 2.**
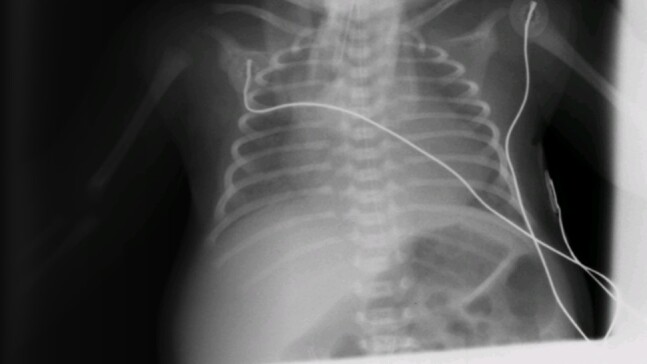
Chest x-ray of the patient. General thinning of the bones are presented on the chest x-ray done shortly after his birth.

Our patient underwent extensive investigations both in the neonatal period and at 18 months of age (sweat test, metabolic and infectious disease screens, karyotype – all normal). His initial skeletal survey showed general thinning of the bones (see Fig. 2 chest x-ray). Genetic testing completed at 3 years of age revealed a pathogenic variant (c.3379-3380 del TG) in exon 18 of the *CUL*7 consistent with 3-M syndrome. The pathogenic variant was present in a homozygous state and led to amino acid change (W1127E) and a premature stop codon (+39, exon 19). Both parents were found to be heterozygous carriers for the same variant.

## Treatment

**Figure 3.**
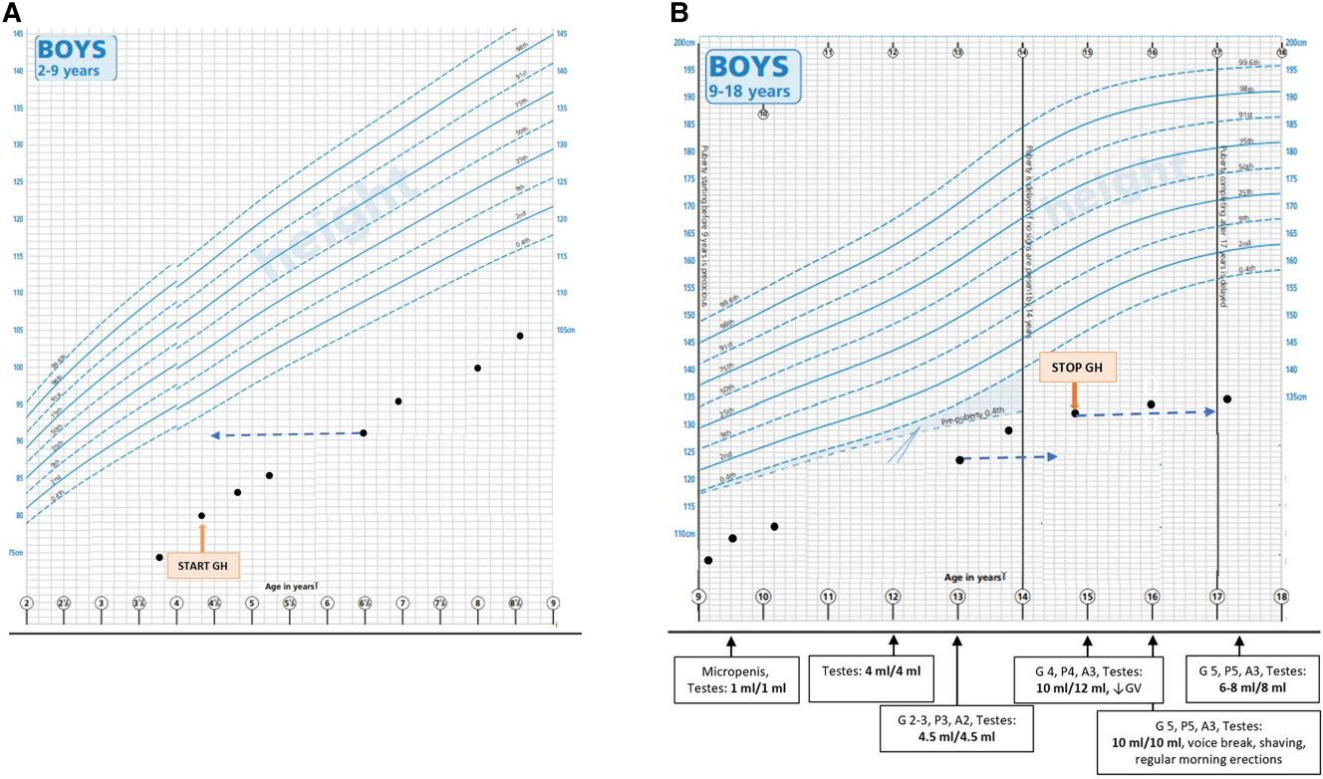
Growth chart. (A) Growth chart at age 2-9 years. (B) growth chart at age 9-18 years. Dashed lines indicate bone age, direction of arrow represents an indication of delay or advancement compared with chronological age. Orange text boxes indicate start or end of rhGH treatment. Along the y-axis of Fig 3B is pubertal staging at different time points.

At the age of 4.5 years, he was started on rhGH at the dose of 0.5 mg once daily (= 52 mcg/kg per day) based on the small-for-gestational-age licensed indication (Fig. 3A). Treatment dose was adjusted according to IGF-1 levels and height velocity (HV). His IGF-1 levels on treatment were above the normal range but rhGH treatment was only adjusted when >2 the times upper limit of normal. rhGH treatment was stopped at age 14.9 years when his HV was 1.6 cm per year (Fig. 3B).

Our patient was diagnosed with obesity at 6.5 years and his BMI SDS reached the maximum at 9 years of age (BMI SDS +3.8, BMI 27.7 kg/m^2^). At 16 years of age (BMI 26.6 kg/m^2^, SDS BMI +1.7), he reduced his weight and has maintained in the overweight category since then.

Our patient underwent first-stage repair of his hypospadias at age 2 years 8 months, second-stage repair at 3 years 4 months, and repair of a small fistula in the penoscrotal region later the same year. For his increased body weight, he has been followed by a dietitian locally and managed to decrease his weight by reducing food intake and making healthy food choices.

## Outcome and Follow-up

*Linear growth:* Our patient´s growth remained far below the third percentile (Fig. 3B). He reached his near-adult height of 136.2 cm (−5.2 SDS) at 17.4 years. He suffered significant bullying and self-confidence issues but made satisfactory progress with clinical psychology support.

*Puberty:* At the age of 9.5 years, he was noted to have micropenis. He spontaneously entered puberty at 12 years of age (Fig. 3B). He attained maximum testicular volumes of 10 to 12 mL at approximately 15 years of age. Subsequently, his testicular volumes had declined to 6 to 8 mL by 17.5 years. He presented with elevated gonadotropins with a slightly low, but still within the normal range, testosterone concentration (Table 1). Furthermore, his inhibin B levels were low (<100 pg/mL; <100 ng/L). Serum inhibin B levels were analyzed using the inhibin B Gen II enzyme-linked immunosorbent assay kit (Beckman Coulter). There are no normal ranges given for this assay clinically but the literature suggests an inhibin B level <100 pg/mL (<100 ng/L) would be considered low (8). His additional androgen profile was normal.

**Table 1.** Anthropometry and laboratory parameters

Age (y)	0.4 y	1 y	1.5 y	4.3 y*^a^*	6.9 y	9.6 y	10.3 y	13 y	14.8 y*^b^*	16.1 y	16.8 y	17.3 y
Tanner stages of puberty						A1, P1, G1 both testes1 mL		A2, P3, G2-3, both testes 4-5 mL	A3, P4, G4, right testes 10 mL, left testes 12 mL	A3, P5, G5, both testes 10 mL		A5, P5, G5, right testes 6-8 mL, left testes 8 mL
Height (cm) SDS	47.0 -8.7	57.0 -7.9	60.2 -8.2	81.4 -5.5	97.1 -4.6	111.1 -4.0	113.7 -3.9	127.8 -3.8	133.7 -4.4	135.2 -4.9	—	136.2 -5.2
HV (cm/year)		—	6.4	—	8.4	5.4	3.9	—	1.5	1.2	—	—
Weight (kg) SDS	3.54 -6.4	5.6 -4.9	6.2 -5.1	9.6	18.6	33.8 0.7	35.9 0.7	46.5 0.2	53.1 -0.3	48.6 -1.6	—	52.4 -1.6
BMI (kg/m^2^) SDS	16.0 -0.9	17.2 0.3	17.0 0.6	14.5 -0.6	19.7 2.3	27.4 3.5	27.8 3.3	28.7 2.7	29.7 2.5	26.6 1.7	—	28.2 1.9
Testosterone NR 201.7-778.1 ng/dL (NR 7.0-27.0 nmol/L)	—	—	—	—	—	—	—	—	276.7 ng/dL (9.6 nmol/L)	293.9 ng/dL (10.2 nmol/L)	371.8 ng/dL (12.9 nmol/L)	340 ng/dL (11.8 nmol/L)
LH NR 0.7-9.1 mIU/mL (NR 0.7-9.1 IU/L)	—	—	—	—	—	—	—	—	**11.5 mIU/mL** **(11.5 IU/L)**	**15.0 mIU/mL** **(15 IU/L)**	**11.3 mIU/mL** **(11.3 IU/L)**	**13.1 mIU/mL** **(13.1 IU/L)**
FSH NR 1.0-11.0 mIU/mL) (NR 1.0-11.0 IU/L)	—	—	—	—	—	—	—	—	**24.9 mIU/mL** **(24.9 IU/L)**	**21.8 mIU/mL** **(21.8 IU/L)**	**16.5 mIU/mL** **(16.5 IU/L)**	**16.4 mIU/mL** **(16.4 IU/L)**
AMH NR 5.6-78.5 ng/mL (NR 40.0-560.8 pmol/L)	—	—	—	—	—	—	—	—	—	—	**4.3 ng/mL** **(30.8 pmol/L)**	**4.4 ng/mL** **(31.3 pmol/L)**
Inhibin B NR >100 pg/mL*^c^* (NR >100 ng/L)	—	—	—	—	—	—	—	—	—	—	**61.6 pg/mL** **(61.6 ng/L)**	**50.7 pg/mL** **(50.7 ng/L)**

Tanner stages of puberty: A, evaluation of axillary hair growth; P, pubic hair growth; G, genitalia growth. Values in parentheses are International System of Units (SI). Abnormal values are shown in bold font.

Abbreviations: AMH, anti-Müllerian hormone; BMI, body mass index; HV, height velocity; NR, normal range (age- and gender-specific laboratory values); SDS, SD score.

^*a*^Start GH treatment.

^*b*^Stop GH treatment.

^*c*^No available reference range for the essay used, value <100 pg/mL is considered abnormal (8).

## Discussion

Our patient presented with prenatal and postnatal severe growth restriction. He had a normal GH response in the GH stimulation test but was eligible for rhGH treatment in the United Kingdom under the small-for-gestational-age license. He was treated for 11 years and showed a degree of catch-up growth (−5.5 SDS to −3.8 SDS) by his 13th birthday. However, the rhGH led to normalization of HV only within the first 3.5 years of treatment, which is in line with previous case reports (4). Because of high IGF-I concentrations, our patient´s GH dose had to be decreased throughout the treatment. Additionally, there was only a minimal pubertal growth spurt. His near-adult height of 136.2 cm (−5.2 SDS) is far below minimal parental height prediction. The contribution of rhGH to our patient's growth is thus debatable. The use of rhGH or rhIGF-1 for short stature in patients with 3-M syndrome typically shows a modest response (1). Second, patients exhibit variable efficacy of GH treatment partly from genotype differences (6). Deeb et al reported a good response to GH therapy in a *CUL7* pathogenic variant carrier (8), but in general, individuals with *CUL7* pathogenic variants are significantly shorter than those with *CCDC8* or *OBSL1* pathogenic variants (1).

Our patient underwent surgery for perineal hypospadias during early childhood. At 9.5 years he was noted to have a micropenis (stretched penile length 3.5 cm, <3rd percentile) with 1 mL volume testes bilaterally. No treatment was given at this time. He entered puberty spontaneously at 12 years and continued until 15 years of age. At this time, his HV had slowed down to 1.6 cm/year. At aged 17 years, he was noted to have testicular volume regression (6-8 mL testes from 10-12 mL testes aged 15 years) with an unstretched penile length of 7.5 cm. Although he was still experiencing morning erections at this time with low-normal adult male testosterone levels, his FSH and LH were elevated (Table 1).

Hypogonadism with or without hypospadias has been demonstrated in a few males with this syndrome (2, 9). This is the first case report in 3-M syndrome to also report on inhibin B and anti-Müllerian hormone levels. Lower levels of inhibin B have been shown in research studies to be a marker of Sertoli cell function and spermatogenesis, although the range in normospermic males is highly variable and cannot be taken in isolation (10). Only 1 case of a male *CUL7* pathogenic variant carrier with hypergonadotropic hypogonadism has so far been reported (11). This male was diagnosed in adolescence and did not show other features of hypogonadism. It is still unclear whether this is found only in *CUL7* pathogenic variant carriers. *CUL7* pathogenic variant carriers in the Yakuts population did not demonstrate hypogonadism, and hypospadias was observed only in 1 case (7). No data on fertility are available apart from a study on the Yakut population that reported offspring with normal height in 2 men and 1 woman with 3-M syndrome caused by *CUL7* pathogenic variants (7).

It is important to mention the possible psychological impact of short stature, such as bullying, low self-esteem, and other psychological disorders, in individuals with this condition. A routine psychology screening and adaptive aids should be offered. Finally, patients with 3-M syndrome may require long-term dietician counseling—first for failure to thrive, later for higher risk of overweight and obesity. Interestingly, a downregulation of leptin in 3-M syndrome was found by Murray et al, who suggested this as a mechanism to increase energy intake to promote growth (5). It will be interesting to see whether antiobesity medicines could be used in these patients. Individuals with 3-M syndrome require a multidisciplinary approach during childhood, adolescence, and adulthood. Issues related to quality of life, fertility, and body weight should be addressed at adult follow-up clinics.

This case highlights the importance of regular reproductive endocrine axis evaluation as well as growth assessment in 3M in childhood etc.

## Learning Points

- Pubertal function needs to be assessed in all individuals with 3 M syndrome.
- rhGH treatment may only offer limited value to increasing adult height and should be reviewed on an annual basis.
- Families should be counseled on the possible limited value of rhGH at the time of initiation of treatment.

## Contributors

I.A.H. and E.S.B. collected and analyzed the results. E.K. reviewed genetic data. I.A.H. and R.P.D. had full access to all the data in the study and are responsible for the final article. I.A.H. wrote the manuscript. R.P.D. conceived the article, reviewed and revised the manuscript, and had final responsibility for the decision to submit it for publication.

## Data Availability

Original data generated and analyzed for this case report are included in this published article.
